# Supplementary material for: Combined Radionuclide Therapy and Immunotherapy for Treatment of Triple Negative Breast Cancer
Source: Int J Mol Sci. 2021 May 3;22(9):4843. doi: 10.3390/ijms22094843 (PMC8124136; doi:10.3390/ijms22094843)
Supplement: Supplementary file 1 [file ijms-22-04843-s001.zip › ijms-1196150-supplementary.pdf]

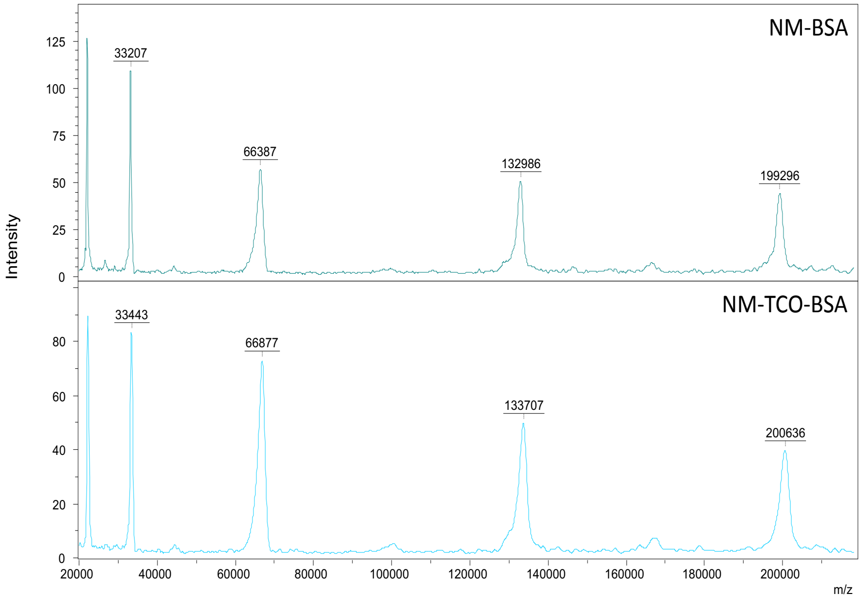

Figure S1. MALDI-MS confirms conjugation of TCO to BSA. BSA (top spectrum) was conjugated with TCO (bottom spectrum) and analyzed via MALDI-MS, indicating an average of 3.2 TCO groups per BSA.

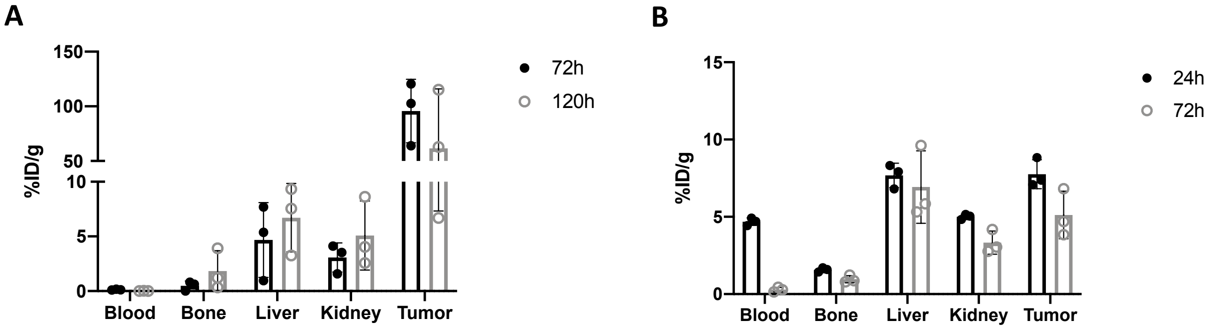

Figure S2. Biodistribution studies show retention of RT in the tumor with intratumoral administration. (**A**) Balb/c mice bearing subcutaneous 4T1 tumors were administered 0.22 MBq (SD ± 0.16 MBq) intratumorally. Groups of mice (n = 3) were sacrificed 72 and 120 h after treatment, and tissues were harvested to assess tumor retention and uptake in non-target tissues. (**B**) Balb/c mice bearing subcutaneous 4T1 tumors were administered 0.22 MBq (SD ± 0.16 MBq) intravenously. Groups of mice (n = 3) were sacrificed 24 and 72 h after treatment, and tissues were harvested to assess tumor retention and uptake in non-target tissues.

Table S1. Biodistribution studies, intratumoral administration. %ID/g values for the complete tissue list for each mouse (n = 3 per timepoint).

Tissue	72 h			120 h		
Blood	0.18	0.12	0.10	0.01	0.03	0.01
Adipose	0.00	0.58	0.55	0.71	1.21	0.45
Adrenals	0.00	0.97	0.83	2.32	3.18	0.87
Bone	0.00	0.63	0.82	1.22	3.92	0.34
Brain	0.00	0.02	0.01	0.01	0.12	0.04

Gall Bladder	0.00	1.88	0.00	6.82	6.99	0.60
Heart	0.00	0.73	1.02	1.37	1.40	0.34
Kidneys	1.58	3.53	4.11	4.05	8.63	2.56
Large Intestine + Caecum	0.26	0.47	0.60	0.50	0.75	0.32
Liver	0.96	5.37	7.70	7.55	9.34	3.25
Lungs	0.00	0.61	1.03	0.84	1.08	0.21
Pancreas	0.00	0.66	0.90	0.87	1.02	0.23
Skeletal Muscle	0.00	0.39	0.55	6.46	18.85	0.25
Small Intestine	0.19	0.35	0.49	0.41	0.55	0.11
Spleen	0.00	2.27	3.46	2.57	3.69	0.70
Stomach	0.32	0.26	0.45	0.64	0.48	0.14
Thyroid/Trachea	0.00	0.67	1.17	0.69	1.23	1.08
Tumor	63.95	102.63	120.56	6.69	115.25	62.89
Urine + Bladder	2.18	8.79	0.94	4.68	11.09	2.60

Table S2. Biodistribution studies, intravenous administration. %ID/g values for the complete tissue list for each mouse (n = 3 per timepoint).

**Tissue**		**24 h**			**72 h**	
Blood	4.70	4.92	4.43	0.44	0.15	0.28
Adipose	1.24	1.63	1.31	0.84	1.05	1.23
Adrenals	5.65	3.58	4.69	2.75	2.29	3.79
Bone	1.70	1.59	1.43	0.87	0.79	1.23
Brain	0.09	0.09	0.09	0.02	0.02	0.02
Gall Bladder	5.67	2.36	9.61	0.92	1.43	5.33
Heart	2.68	2.27	2.93	1.40	1.18	1.93
Kidneys	4.83	5.04	5.15	2.77	3.05	4.18
Large Intestine + Caecum	1.14	0.97	1.01	0.66	0.44	1.00
Liver	7.92	6.80	8.32	5.85	5.32	9.62
Lungs	2.30	1.93	2.70	0.87	0.71	1.30
Pancreas	2.74	1.22	1.50	0.87	0.82	1.27
Skeletal Muscle	0.91	1.27	0.96	0.39	0.67	0.66
Small Intestine	1.17	0.84	0.99	0.44	0.31	0.62
Spleen	5.74	4.32	4.77	2.68	1.97	4.33
Stomach	0.72	0.64	0.68	0.41	0.39	0.76
Thyroid/Trachea	1.84	2.00	2.24	0.79	1.05	1.35
Tumor	7.08	8.83	7.37	3.84	4.69	6.82
Urine + Bladder	4.96	2.95	5.12	3.75	3.30	1.76

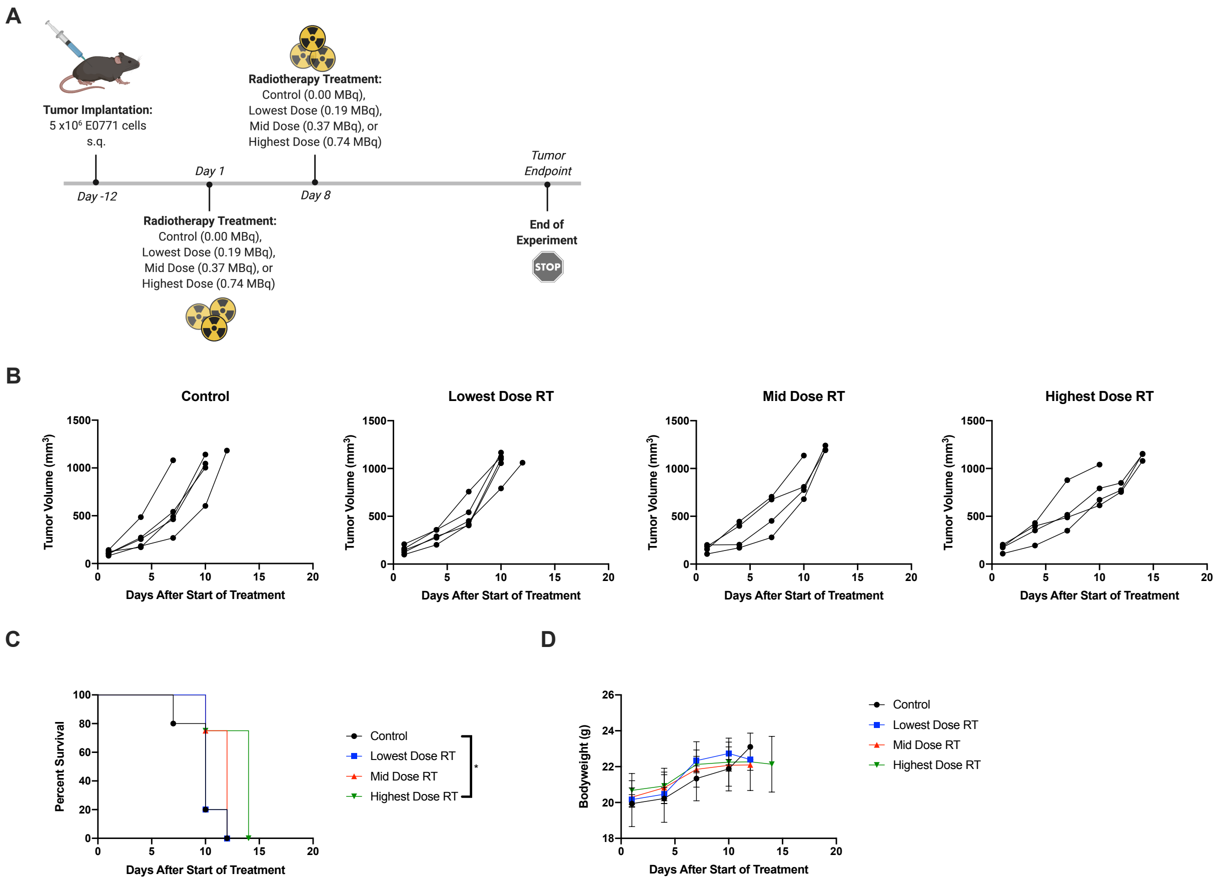

Figure S3. Preliminary dose optimization studies for RT regimens. (**A**) C57/Bl6 mice bearing E0771 tumors were treated with control, lowest dose RT, mid dose RT, or highest dose RT. Each treatment schedule outlines a separate experiment. (**B**) Tumor volumes were measured every 2–3 days from the start of treatment until mice reached endpoint. Each line represents an individual mouse within the group. (**C**) Kaplan–Meier survival curves of each group. (**D**) Average bodyweights for all groups.

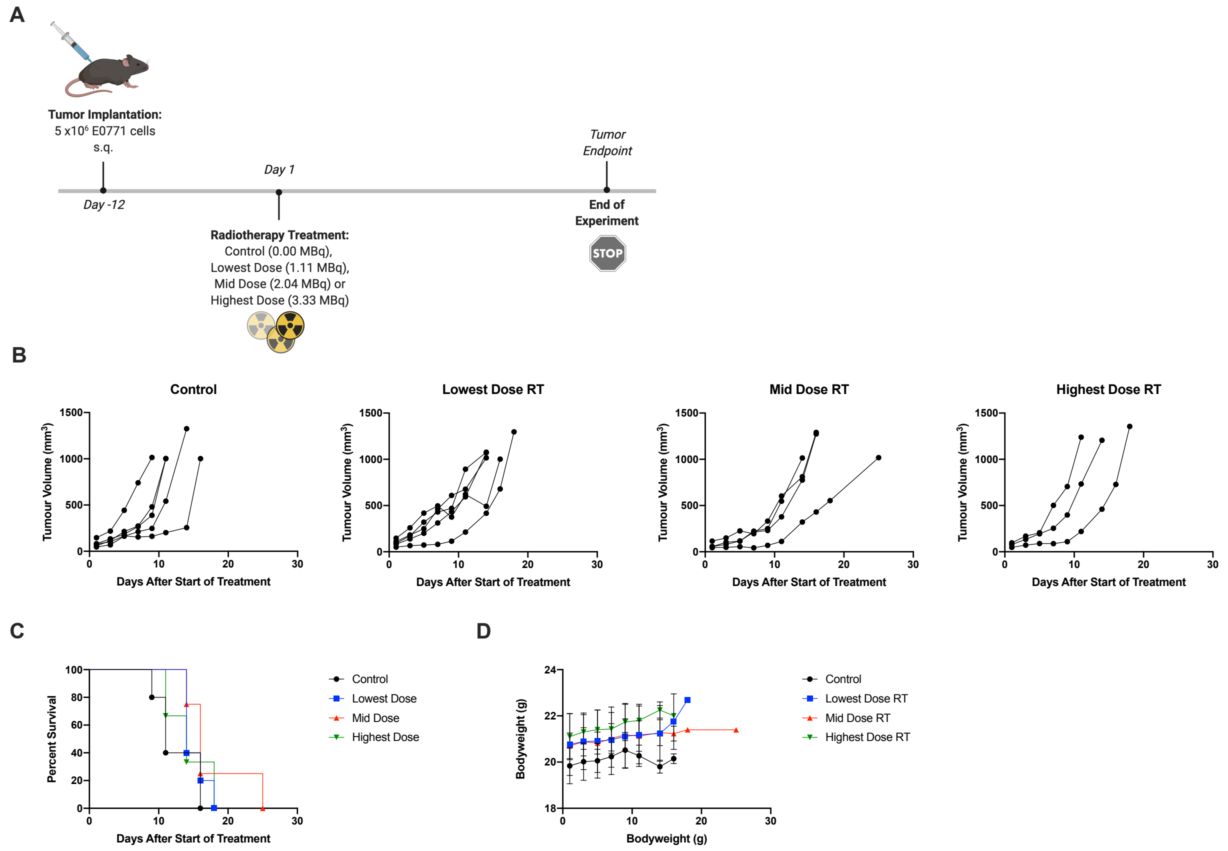

Figure S4. Dose optimization studies for RT regimens. (**A**) C57/Bl6 mice bearing E0771 tumors were treated with control, lowest dose RT, mid dose RT, or highest dose RT. Each treatment schedule outlines a separate experiment. (**B**) Tumor volumes were measured every 2–3 days from the start of treatment until mice reached endpoint. Each line represents an individual mouse within the group. (**C**) Kaplan–Meier survival curves of each group. (**D**) Average bodyweights for all groups.
